# Effects of an Exercise Program on Cardiometabolic and Mental Health in Children With Overweight or Obesity: A Secondary Analysis of a Randomized Clinical Trial

**DOI:** 10.1001/jamanetworkopen.2023.24839

**Published:** 2023-07-27

**Authors:** Jairo H. Migueles, Cristina Cadenas-Sanchez, David R. Lubans, Pontus Henriksson, Lucia V. Torres-Lopez, María Rodriguez-Ayllon, Abel Plaza-Florido, Jose J. Gil-Cosano, Hanna Henriksson, María Victoria Escolano-Margarit, José Gómez-Vida, José Maldonado, Marie Löf, Jonatan R. Ruiz, Idoia Labayen, Francisco B. Ortega

**Affiliations:** 1 Department of Physical Education and Sports, Faculty of Sport Sciences, Sport and Health University Research Institute, University of Granada, Granada, Spain; 2 Department of Biosciences and Nutrition, Karolinska Institutet, Stockholm, Sweden; 3 CIBER (Centro de Investigación Biomédica en Red) de Fisiopatología de la Obesidad y Nutrición, Instituto de Salud Carlos III, Granada, Spain; 4 Department of Cardiology, Stanford University, Stanford, California; 5 Palo Alto Health Care System, Veterans Affairs Medical Center, Palo Alto, California; 6 Centre for Active Living and Learning, Hunter Medical Research Institute, University of Newcastle, Newcastle, Australia; 7 Faculty of Sport and Health Sciences, University of Jyväskylä, Jyväskylä, Finland; 8 Department of Health, Medicine and Caring Sciences, Linköping University, Linköping, Sweden.; 9 Department of Epidemiology, Erasmus University Medical Center Rotterdam, Rotterdam, the Netherlands; 10 Pediatric Exercise and Genomics Research Center, Department of Pediatrics, School of Medicine, University of California at Irvine; 11 Department of Communication and Education, Universidad Loyola Andalucía, Dos Hermanas, Sevilla, Spain; 12 Department of Pediatrics. San Cecilio University Hospital, Granada, Spain.; 13 Department of Pediatrics, School of Medicine, University of Granada, Granada, Spain; 14 Instituto de Investigación Biosanitaria de Granada, Granada, Spain; 15 Institute for Sustainability & Food Chain Innovation, Navarra Institute for Health Research, Department of Health Sciences, Public University of Navarra, Pamplona, Spain

## Abstract

**Importance:**

Childhood obesity is a risk factor associated with type 2 diabetes, cardiovascular disease, and mental disorders later in life. Investigation of the parallel effects of a defined exercise program on cardiometabolic and mental health in children with overweight or obesity may provide new insights on the potential benefits of exercise on overall health.

**Objective:**

To investigate the effects of a 20-week exercise program on cardiometabolic and mental health in children with overweight or obesity.

**Design, Setting, and Participants:**

This secondary analysis of a parallel-group randomized clinical trial was conducted in Granada, Spain, from November 1, 2014, to June 30, 2016. Data analyses were performed between February 1, 2020, and July 14, 2022. Children with overweight or obesity aged 8 to 11 years were eligible, and the study was performed in an out-of-school context.

**Intervention:**

The exercise program included 3 to 5 sessions/wk (90 min/session) of aerobic plus resistance training for 20 weeks. The wait-list control group continued with their usual routines.

**Main Outcomes and Measures:**

Cardiometabolic outcomes as specified in the trial protocol included body composition (fat mass, fat-free mass, and visceral adipose tissue), physical fitness (cardiorespiratory, speed-agility, and muscular), and traditional risk factors (waist circumference, blood lipid levels, glucose levels, insulin levels, and blood pressure). Cardiometabolic risk score (*z* score) was calculated based on age and sex reference values for levels of triglycerides, inverted high-density lipoprotein cholesterol, and glucose, the mean of systolic and diastolic blood pressure, and waist circumference. An additional cardiometabolic risk score also included cardiorespiratory fitness. Mental health outcomes included an array of psychological well-being and ill-being indicators.

**Results:**

The 92 participants included in the per-protocol analyses (36 girls [39%] and 56 boys [61%]) had a mean (SD) age of 10.0 (1.1) years. The exercise program reduced the cardiometabolic risk score by approximately 0.38 (95% CI, −0.74 to −0.02) SDs; decreased low-density lipoprotein cholesterol level by −7.00 (95% CI, −14.27 to 0.37) mg/dL (to convert to mmol/L, multiply by 0.0259), body mass index (calculated as weight in kilograms divided by height in meters squared) by −0.59 (95% CI, −1.06 to −0.12), fat mass index by −0.67 (95% CI, −1.01 to −0.33), and visceral adipose tissue by −31.44 (95% CI, −58.99 to −3.90) g; and improved cardiorespiratory fitness by 2.75 (95% CI, 0.22-5.28) laps in the exercise group compared with the control group. No effects were observed on mental health outcomes.

**Conclusions and Relevance:**

In this secondary analysis of a randomized clinical trial, an aerobic plus resistance exercise program improved cardiometabolic health in children with overweight or obesity but had no effect on mental health.

**Trial Registration:**

ClinicalTrials.gov Identifier: NCT02295072

## Key Points

**Question:**

What are the parallel effects of exercise on cardiometabolic and mental health in children with an excess of adiposity?

**Findings:**

In this secondary analysis of a parallel-group randomized clinical trial of 92 children with overweight or obesity, a 20-week exercise program including aerobic plus resistance training improved body composition, cardiorespiratory fitness, and cardiometabolic risk factors. No effects on mental health were observed.

**Meaning:**

These findings suggest that exercise programs should be promoted in children with excess body weight to improve their cardiometabolic health.

## Teaser

This secondary analysis of a randomized clinical trial investigates the effects of a 20-week exercise program on cardiometabolic and mental health among children with overweight or obesity.

## Introduction

Obesity is a major risk factor for type 2 diabetes and cardiovascular disease (CVD).^1,2,3^ Best practices for prevention of type 2 diabetes and CVD should start in childhood.^4,5^ Other comorbidities associated with pediatric obesity include poor cardiometabolic^6,7,8^ and mental health.^9^ Exercise is considered an essential component of obesity treatment programs in children due to its physical, psychological, and cognitive benefits.^10^

Previous trials in children with obesity have demonstrated exercise-induced improvements in visceral fat,^11,12^ high (HDL)- and low-density lipoprotein (LDL) cholesterol levels,^11,13^ insulin resistance,^12^ blood pressure,^14^ body composition,^12,13^ cardiorespiratory fitness (CRF),^12,13,14^ and self-worth.^15^ A 2018 scoping review on the topic^16^ stated that measuring cardiometabolic risk as a composite or clustered score that includes measures of adiposity, lipid levels, metabolism, and blood pressure at childhood is a better predictive factor associated with CVD in young adulthood than other categorical measures (eg, the presence of metabolic syndrome).^17^ Likewise, cardiometabolic risk scores have proven to be a better marker of cardiovascular health in children than single risk factors.^18^ However, the parallel effects have not been studied in children, as noted in a systematic review and meta-analysis.^19^ Therefore, understanding the holistic benefits of exercise based on the “polypill” concept of exercise^20^ are needed.

A recent consensus statement called attention to the relevance of exploring and understanding the exercise response variability,^21^ yet information is limited on the individual variability of exercise effects in children with obesity.^22^ Therefore, the aim of this study was to investigate the effects of a 20-week exercise program on cardiometabolic and mental health in children with overweight or obesity. Further, we examine the within-individual change in the effects observed.

## Methods

### Study Design

The ActiveBrains randomized clinical trial (RCT) investigated the effects of exercise on brain and cognitive function in children with overweight and obesity.^23,24^ The trial protocol is found in Supplement 1. This study presents the effects on secondary outcomes from the ActiveBrains RCT; the primary outcomes study and main effects can be found elsewhere.^23^ The ActiveBrains RCT was approved by the Human Research Ethics Committee of the University of Granada. Written informed consent was obtained from the parents or the legal guardians of all participants, who provided informed assent. The study followed the Consolidated Standards of Reporting Trials (CONSORT) reporting guideline.

### Participants

Prepubertal children (aged 8-11 years) with overweight or obesity and not presenting with any neuropsychological (including attention-deficit/hyperactivity disorder) or physical problems were eligible to participate in the ActiveBrains RCT (Supplement 1). We collected country of origin of the parents and the child. More than 90% were categorized as White; therefore, we did not use the race or ethnicity as an important covariate in our study. Recruitment occurred mainly at the hospitals. Data were collected from November 1, 2014, to June 30, 2016.

### Randomization and Masking

Participants were randomly assigned to either the exercise program or the wait-list control group with simple random allocation in a ratio of 1:1 by a blinded individual (F.B.O.). Randomization was performed immediately after the baseline evaluation, and the physical trainers running the exercise program were not involved in the outcome evaluations or randomization.

### Procedures and Interventions

The exercise program had a duration of 20 weeks and was based on the global physical activity recommendations for children, including aerobic and muscle-bone–strengthening activities (hereafter referred to as resistance exercise).^25^ The exercise group was instructed to attend at least 3 (of 5 offered) supervised sessions per week. Each session lasted 90 minutes (60 minutes of aerobic plus 30 minutes of resistance exercise). Heart rate monitors (Polar RS300X; Polar Electro Oy Inc) were used to track participants’ exercise intensity during sessions. Children spent a mean of 38 minutes per session above 80% of their maximum heart rate. Participants in the control group continued with their usual routines. Both control and exercise groups received a pamphlet with general information about healthy nutrition and physical activity recommendations at the beginning of the study. Detailed information can be found in Supplement 1.

### Outcome Measures

Measurements were conducted at baseline and immediately after the end of the intervention. Sociodemographic data were reported by children and their parents. At baseline, somatic maturation was assessed with the peak height velocity.^26^

### Cardiometabolic Health

Cardiometabolic health outcomes included traditional risk factors for cardiometabolic risk score (ie, hyperglycemia, hypertension, and dyslipidemia),^24^ as well as body composition and physical fitness, which are closely related to cardiometabolic health.^27,28^ Blood lipid biomarkers included fasting LDL and HDL cholesterol and triglyceride levels. The ratio of triglyceride to HDL cholesterol levels was calculated. Fasting insulin and glucose levels were obtained from blood samples, and the homeostatic model assessment index was calculated as insulin level (in microunits per milliliter) multiplied by glucose level (in milligrams per deciliter) and divided by 405. All blood samples were collected at the hospital after a minimum of 8 hours of overnight fasting. Systolic and diastolic blood pressure were assessed twice in a sitting position from the left arm with an automatic sphygmomanometer (M6; Omron), and the lowest values were considered. The mean systolic and diastolic blood pressure and the mean arterial pressure were calculated. Then, the risk of dyslipidemia (ie, alteration of triglyceride and/or HDL cholesterol levels), prediabetes (glucose level), and prehypertension (systolic and diastolic blood pressure) were classified based on age- and sex-specific cutoffs.^29^

Body weight and height were measured twice using an electronic scale and stadiometer (Seca GmbH). Body mass index was calculated as weight in kilograms divided by height in meters squared and was used to derive the age- and sex-specific *z* scores according to World Health Organization references.^30^ Whole-body fat mass and lean mass and visceral adipose tissue were measured via dual-energy x-ray absorptiometry (Discovery Horizon DXA system; Hologic Inc). Fat mass index and lean mass index were calculated as fat or lean mass in kilograms divided by height in meters squared. Abdominal obesity was represented by the mean waist circumference from 2 measurements.^31^ The following physical fitness components were assessed: CRF using the 20-m shuttle run (laps and estimated maximum oxygen consumption [V̇o_2_max])^32,33^; speed and agility using the 4 × 10-m shuttle run (time to complete the circuit); and muscular fitness using the handgrip (in kilograms) and standing long jump (in centimeters) tests. These tests are valid, reliable, and feasible.^34,35,36^ Detailed information can be found elsewhere.^24^ Then, children with poor fitness were classified based on age- and sex-specific international reference values for CRF in children.^37^

Finally, a previously validated cardiometabolic risk score was calculated.^38^ The score calculated the mean age- and sex-specific *z* scores^39^ for levels of triglycerides, inverted HDL cholesterol, and glucose, the mean of systolic and diastolic blood pressure, and waist circumference.^38^ Since the American Heart Association has recently proposed CRF as a powerful marker cardiometabolic health,^40^ we additionally included CRF performance in a second cardiometabolic risk score. Children at risk of metabolic syndrome were identified as those with a *z* score of at least 0.39 (ie, deviating 0.39 SDs from the European pediatric population) as previously proposed.^39^

### Mental Health

Children completed the mental health questionnaires on 3 nonconsecutive days. Psychological ill-being and well-being components of mental health were assessed using valid self-reported questionnaires. Psychological ill-being measures included stress,^41^ anxiety,^42^ depression,^43^ and negative affect.^44^ Psychological well-being measures included positive affect,^44^ happiness,^45^ optimism,^46^ self-efficacy,^47^ self-concept,^48^ and self-esteem.^49^ A detailed description can be found elsewhere.^24^ Composite standardized scores were calculated for psychological ill-being, psychological well-being, and total mental health (ie, psychological ill-being multiplied by −1 and psychological well-being). Additionally, we calculated the risk of anxiety (cutoff score ≥40 for State-Trait Anxiety Inventory)^50^ and depression (cutoff score ≥19 for Children’s Depression Inventory).^51^

### Physical Activity Assessment

Accelerometer-determined daily time spent in physical activity, sedentary behavior, and sleep during the intervention were used to assess the change in daily activity induced by the exercise intervention. Accelerometers (GT3X+; ActiGraph) were placed on the right hip and the nondominant wrist for 7 days at baseline and during the intervention-delivered period for exercise and control groups. The accelerometer raw data were processed as described elsewhere,^52^ following the practical recommendations previously made by Migueles et al.^53^ We used the GGIR software package (R Project for Statistical Computing)^54^ to identify the night sleep periods using an automated algorithm guided by the self-reported sleep times.^55,56^ Waking time was classified into moderate to vigorous physical activity, light physical activity, and sedentary behavior using children-specific cut points.^57,58,59^

### Statistical Analysis

A posteriori power analysis showed that a sample size of 92 children is enough to detect small-to-medium effect sizes (ie, 0.3 SDs), assuming an α error of .05 and 80% statistical power. Data were analyzed between February 1, 2020, and July 14, 2022. Characteristics of the study participants are presented as mean (SD) or frequency (percentage). Prior to analyses, raw scores from each outcome were winsorized (when needed) to limit the influence of extreme values.^60^ Then, baseline *z* scores of the outcomes were calculated by subtracting their mean and dividing by their SD. Postexercise *z* scores were calculated relative to the baseline mean (SD) as a standardized measure of the effect size.^60^ Analysis of covariance models were built including postexercise outcome values as dependent variables, group (ie, exercise vs control) as a fixed factor, and baseline levels of the outcome studied as covariate.^60^ We conducted the outcome analyses under both the per-protocol (ie, attending ≥70% of the sessions) and the intention-to-treat (ie, including all participants and imputing the missing data using predictive mean matching multiple imputations) principles, following the CONSORT guidelines. Since we primarily aimed to study the efficacy of the program rather than its effectiveness—that is, we wanted to know the association with health outcomes when a child actually performed the planned exercise program (operationally defined as attending a minimum of 70% of the sessions)^61^—we decided to report the per-protocol analyses herein, while the intention-to-treat analyses can be found in eTables 4 and 6 in Supplement 2. The within-individual change distribution was studied and the changes exceeding a Cohen *d* of 0.2 were considered meaningful (accepted threshold for relevant standardized effect size).^22^ We used χ^2^ tests to compare the rate of meaningful changes observed in the exercise and the control groups. Additionally, we explored the change in the daily distribution of the movement behaviors induced by the exercise program. This comparison was performed following the compositional data analysis standards,^62^ in line with the conclusions reported by a previous expert consensus for the analysis of device-measured movement behaviors.^63^ Values in the change composition are represented as proportional changes from the baseline overall composition, and the Hotelling *T*^2^ test for multivariate pairwise comparisons was used. All the statistical procedures were performed using R, version 4.0.0 (R Project for Statistical Computing). The threshold for statistical significance was 2-sided *P* < .05.

## Results

**Figure 1.**
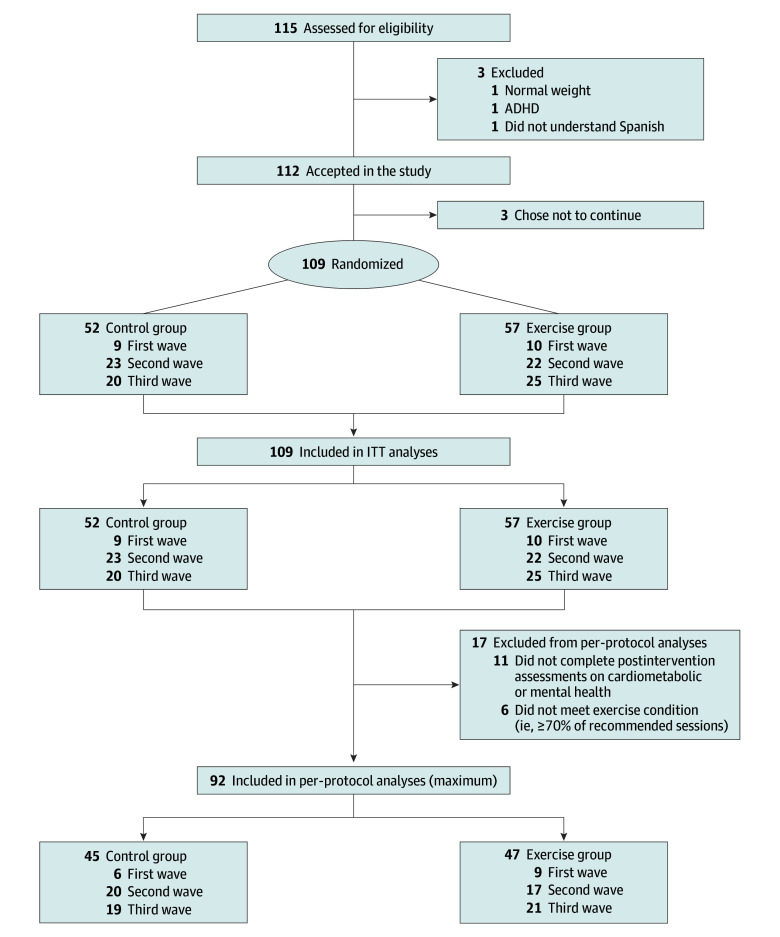
Study Flowchart For final intention-to-treat (ITT) analyses, those participants who left the study or did not complete the postexercise program assessments were imputed. The maximum number for analyses changed depending on the variable (eTables 3-5 in Supplement 2 present the main study outcomes). ADHD indicates attention-deficit/hyperactivity disorder.

Of the 109 participants enrolled, 92 were included in the per-protocol analysis (36 girls [39%] and 56 boys [61%]; mean [SD] age, 10.0 [1.1] years) after excluding those who did not attend the postintervention assessments (n = 11) and those who did not meet the per-protocol criteria (n = 6) (Figure 1). Of the 17 participants who dropped out, 7 were from the control group and 10 were from the exercise group. Participants’ characteristics are presented in eTable 1 in Supplement 2. At baseline, 43 children (44%) were at risk of dyslipidemia, 3 (3%) presented with prediabetes, 10 (10%) presented with prehypertension, 62 (63%) had poor fitness, 76 (77%) had obesity, 25 (25%) were at risk of metabolic syndrome, 18 (19%) were at risk of anxiety, and 3 (3%) were at risk of depression. No significant differences were found regarding baseline characteristics (eg, age, body mass index, fat mass index, parental educational level) between participants who adhered to the protocol (n = 92) and the rest of the participants measured at baseline (n = 17) (eTable 2 in Supplement 2).

### Cardiometabolic Health

**Figure 2.**
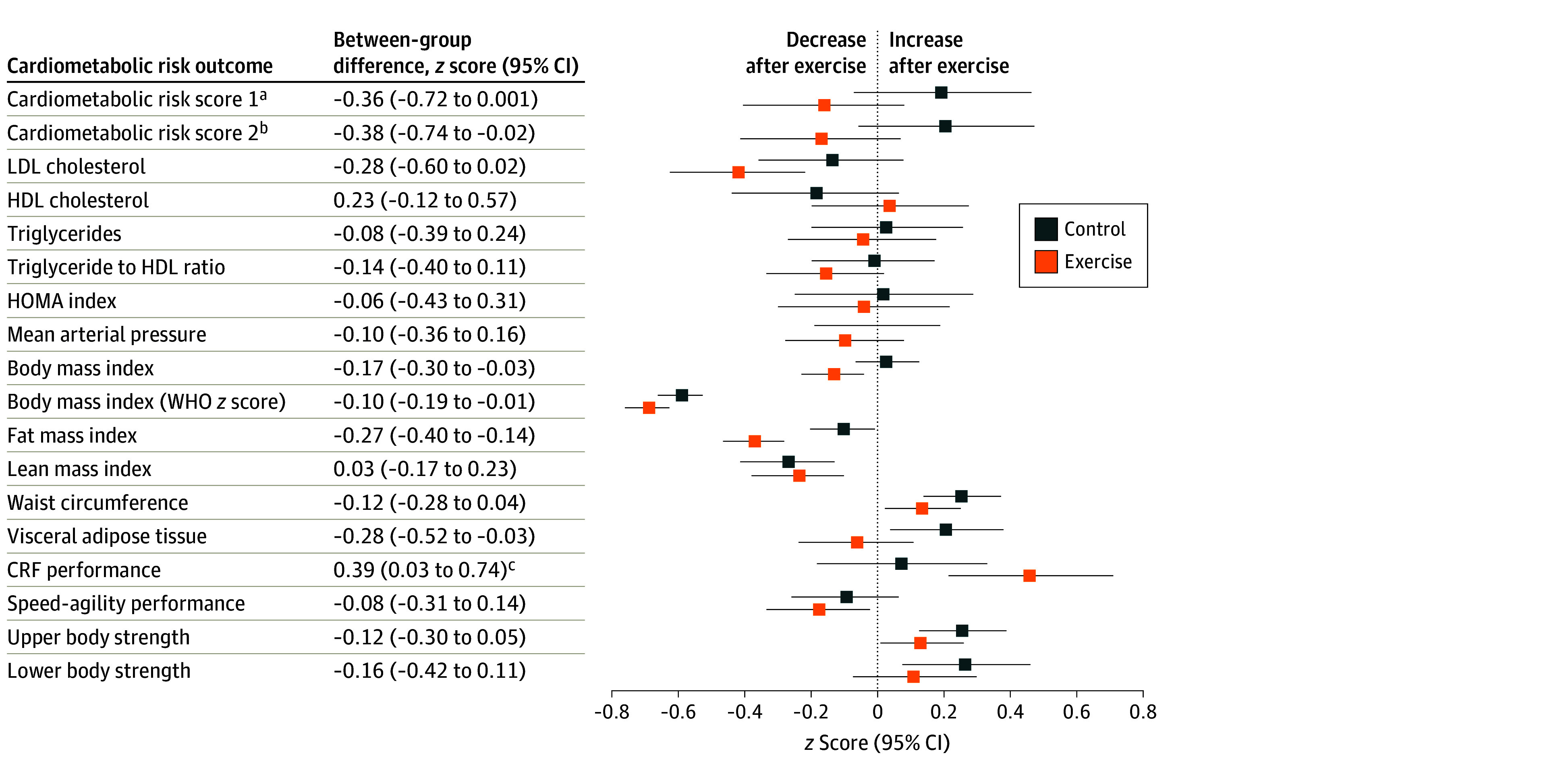
Effects of the Exercise Program *z* Score Change Between Groups in Cardiometabolic Risk Outcomes Data analyses were primarily conducted under the per-protocol principle—that is, participants attending at least 70% of the sessions. Baseline *z* score of the outcomes were calculated by subtracting the mean value and dividing by the SD of each outcome. Postexercise *z* scores were calculated by subtracting the baseline mean and dividing by the baseline SD, being a *z* score of the change in each outcome. HDL indicate high-density lipoprotein; HOMA, homeostatic model assessment; LDL, low-density lipoprotein; and WHO, World Health Organization. ^a^Calculated as the age- and sex-normalized scores for HDL cholesterol level, waist circumference, triglyceride level, glucose level, and the mean of systolic and diastolic blood pressure based on European reference values.^39^ ^b^Calculated as for cardiometabolic risk score 1 and additionally included cardiorespiratory fitness (CRF) as measured by laps in the 20-m shuttle run test. ^c^*P* < .05.

Figure 2 shows the within- and between-groups preintervention-postintervention differences in cardiometabolic health outcomes (tabulated data in eTable 3 in Supplement 2). The exercise program was associated with a reduction in cardiometabolic risk (score 1: −0.36 [95% CI, −0.72 to 0.00] SDs; score 2: −0.38 [95% CI, −0.74 to −0.02] SDs). We found a nonsignificant reduction in LDL cholesterol level of 7.00 (95% CI, −14.27 to 0.37) mg/dL (to convert to mmol/L, multiply by 0.0259) and significant reductions in body mass index (−0.59 [95% CI, −1.06 to −0.12]), fat mass index (−0.67 [95% CI, −1.01 to −0.33]), and visceral adipose tissue (−31.44 [95% CI, −58.99 to −3.90] g) in the exercise group compared with the control group. The exercise group improved CRF performance (2.75 [95% CI, 0.22-5.28] laps) and estimated V̇o_2_max (0.94 [95% CI, 0.05-1.84] mL/kg/min) compared with the control group. Overall, the intention-to-treat analyses showed consistent, but attenuated, effects (eTable 4 in Supplement 2).

**Figure 3.**
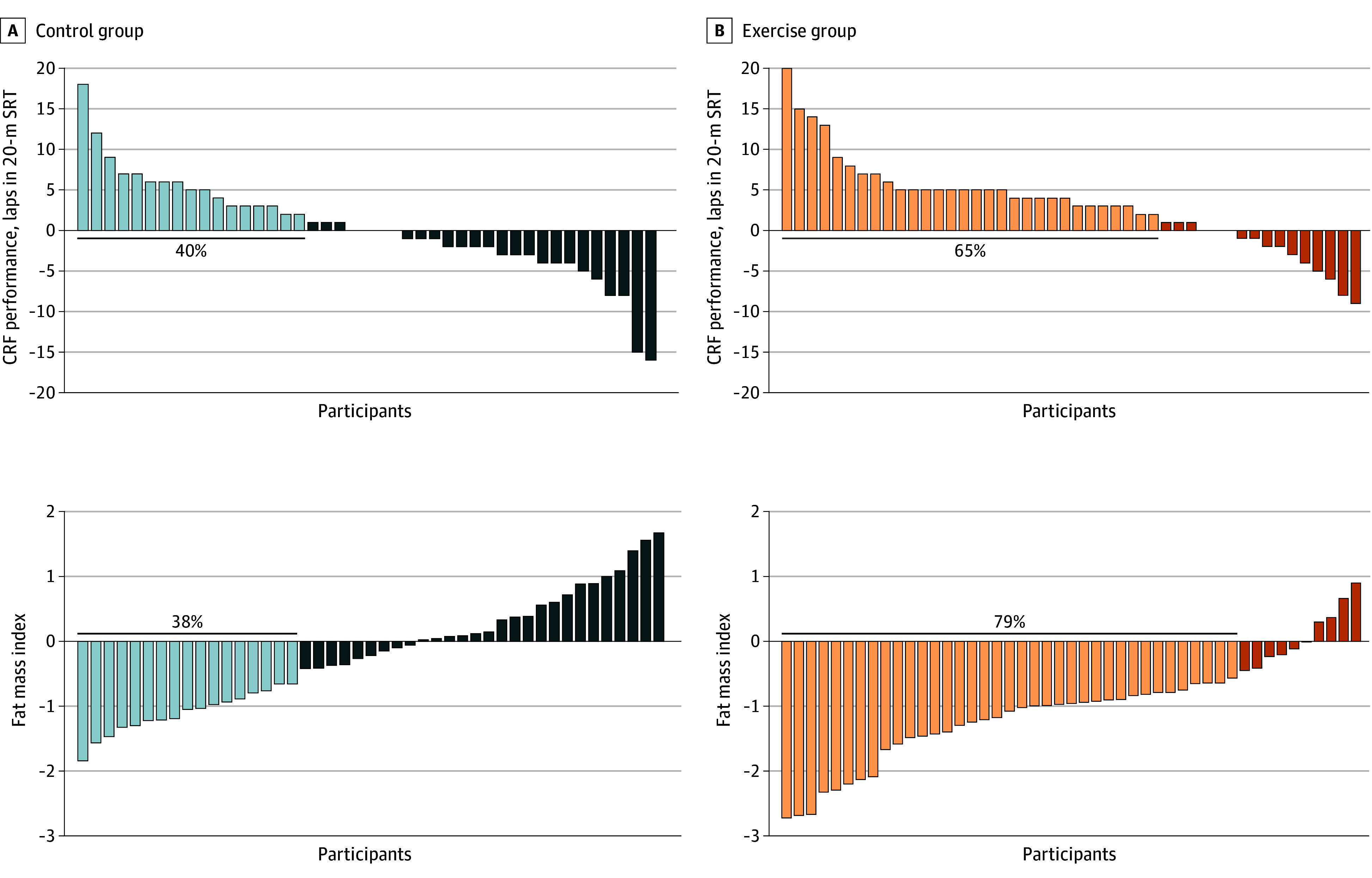
Individual Change Distribution in the Outcomes Significantly Affected by the Exercise Program Data analyses were primarily conducted under the per-protocol principle—that is, participants attending at least 70% of the sessions. CRF indicates cardiorespiratory fitness, measured using the 20-m shuttle run test (20-m SRT). The light blue and light orange bars represent participants who experienced a clinically meaningful change (Cohen *d* = 0.2). The dark blue and dark orange bars represent participants who did not experience a clinically meaningful change or experienced a negative change (Cohen *d* < 0.2).

**Figure 4.**
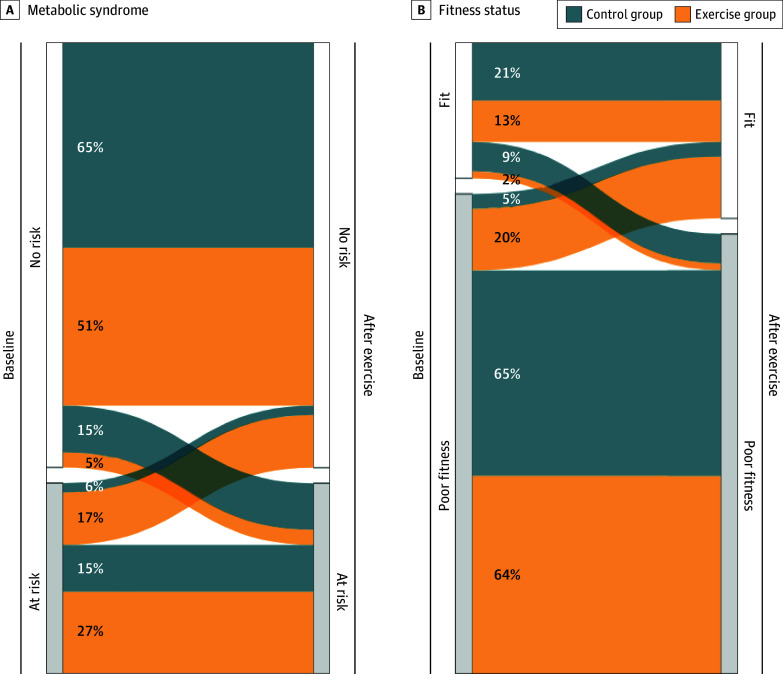
Participant Rate Fluctuations for Risk of Metabolic Syndrome or Fitness Status From Baseline to After Exercise Risk of metabolic syndrome (A) was categorized based on the mean of age- and sex-specific *z* scores for triglyceride levels, inverted high-density lipoprotein cholesterol levels, glucose levels, the mean of systolic and diastolic blood pressure, and waist circumference. Based on European reference values,^39^ those children with a *z* score of 0.39 or greater were considered at risk of metabolic syndrome. Children with poor fitness (fitness status in B) were classified based on age- and sex-specific international reference values for cardiorespiratory fitness in children.^37^

More participants in the exercise group showed meaningful changes (ie, within-individual changes of ≥0.2 SDs) than in the control group in fat mass index (37 [79%] vs 17 [38%]; *P* < .001) and CRF performance (30 [65%] vs 17 [40%]; *P* = .03) (Figure 3). A nonsignificant difference was found in favor of exercise in body mass index (16 [34%] vs 7 [16%]; *P* = .07) (eFigure 1 in Supplement 2). Likewise, we observed that more children at risk of metabolic syndrome at baseline were not at risk after the exercise program in the exercise group compared with the control group (Figure 4A), and a similar trend was observed in children passing from poor fitness to fit status based on cardiorespiratory fitness (9 [20%] vs 2 [5%]) (Figure 4B).

### Mental Health

**Figure 5.**
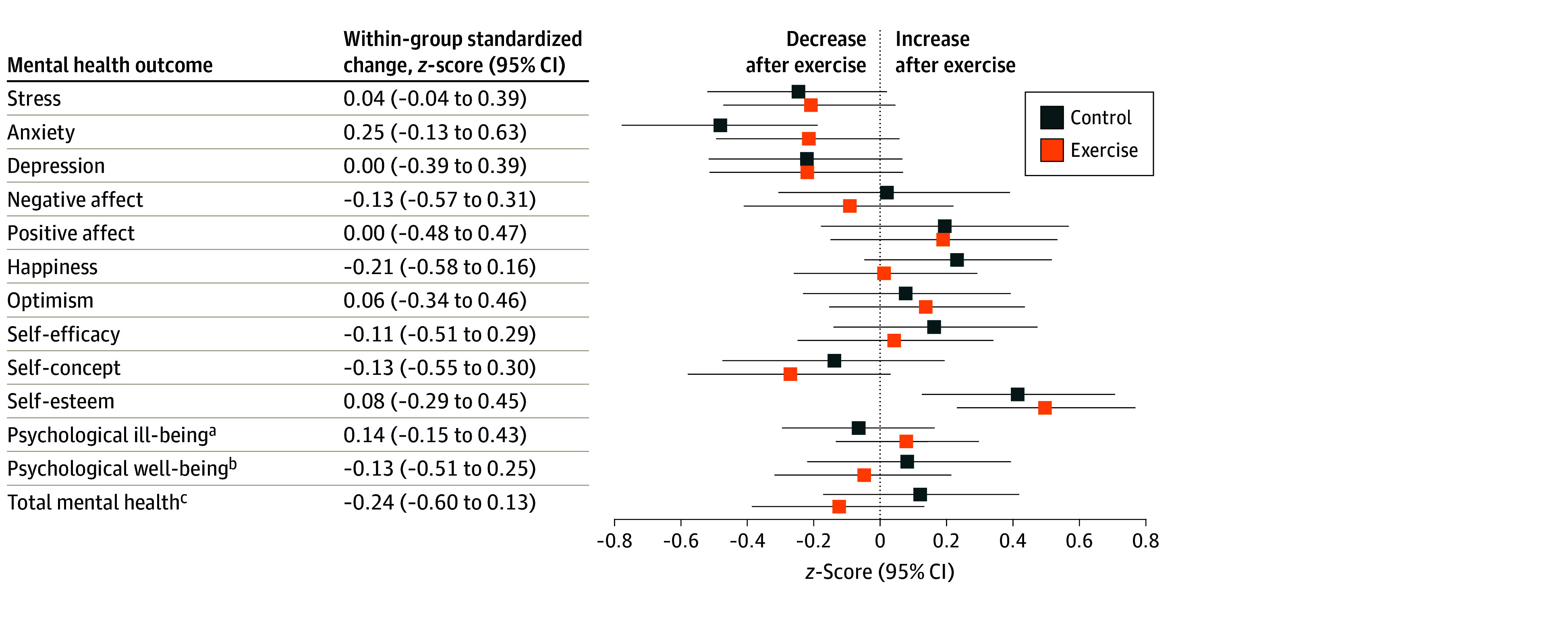
Effects of the Exercise Program *z* Score Change Between Groups in Mental Health Data analyses were primarily conducted under the per-protocol principle—that is, participants attending at least 70% of the sessions. Baseline *z* scores of the outcomes were calculated by subtracting the mean value and dividing by the SD of each outcome. Postexercise *z* scores were calculated by subtracting the baseline mean and dividing by the baseline SD, being a *z* score of the change in each outcome. ^a^Calculated as the normalized mean of the *z* score for stress, anxiety, depression, and negative affect. ^b^Calculated as the normalized mean of the *z* score for positive affect, happiness, optimism, self-efficacy, self-concept, and self-esteem. ^c^Calculated as the normalized mean of the *z* score for all mental health indicators.

Figure 5 shows that the exercise program did not affect any mental health outcome (tabulated data in eTable 5 in Supplement 2). Similarly, intention-to-treat analyses showed no effects on mental health (eTable 6 in Supplement 2).

### Exploratory Analysis: Change in Daily Activity Composition

eFigure 2 in Supplement 2 shows the exercise-induced changes in physical behaviors derived from the hip- and wrist-worn accelerometers. Both the hip- and the wrist-based estimations showed consistent trends that the exercise group increased moderate to vigorous physical activity compared with the control group (hip: +15% vs +7% from baseline; wrist: +21% vs +7% from baseline). More specifically, the wrist-worn estimates resulted in a significant group-by-time effect (*P* = .002) (eFigure 2B in Supplement 2), while the hip-worn estimate was not significant (*P* = .08) (eFigure 2A in Supplement 2). Likewise, the control group did not substantially alter their time in light physical activity, sedentary behavior, and sleep from baseline, while the exercise group substantially reduced their sedentary behavior (hip: −6%; wrist: −14%) and sleep time (hip: −8%; wrist: −9%).

## Discussion

The ActiveBrains RCT demonstrated that a 20-week exercise program reduced the cardiometabolic risk score in children with overweight or obesity. These findings were confirmed using 2 valid cardiometabolic risk scores.^38,40^ Specifically, the exercise program substantially improved children’s body composition (ie, body mass index, fat mass, and visceral fat) and CRF compared with the control group. The proportion of children experiencing meaningful changes in cardiometabolic risk score, body composition, and CRF was higher in the exercise group compared with the control group. No significant effects were observed for the different mental health outcomes.

### Cardiometabolic Health

Our study has demonstrated a sizable reduction in cardiometabolic risk (approximately 0.38 SDs), and the within-individual change showed more participants at risk of metabolic syndrome at baseline were no longer at risk after exercise compared with controls. We reason that the risk reduction was mainly due to the improvements in blood lipid levels, total and visceral adiposity, and CRF, which were the cardiometabolic outcomes affected by the exercise program. In agreement with the American Heart Association,^40^ our study supports the use of CRF as a cardiometabolic risk factor. In addition, the exercise group reduced their fasting LDL cholesterol levels by 7.00 mg/dL (ie, 0.3 SDs) and their visceral adipose tissue by 31.44 g compared with the control group. Other blood lipid and adiposity markers showed a better trend in the exercise compared with the control group, yet did not reach statistical significance (eg, waist circumference and HDL cholesterol level).

Our results are consistent with recent meta-analyses in children with overweight or obesity showing that concurrent aerobic and resistance training can improve blood lipid levels, mainly LDL cholesterol and triglycerides.^64,65^ Our findings are also consistent with previous research showing reductions in visceral fat,^11,12^ LDL cholesterol level,^11^ and increments in HDL cholesterol level following exercise in children with overweight or obesity.^13,14^ Two of the previous RCTs in children with obesity additionally found effects on insulin resistance,^12,14^ yet we did not. We believe that differences in the participants’ baseline characteristics may account for our lack of effects on glucose metabolism biomarkers. For example, a previous RCT analyzed 222 participants, of whom 28% were children with prediabetes,^12^ compared with only 3% in our study. Most of our participants (77%) had obesity, 44% were at risk of dyslipidemia,^29^ and 63% had poor fitness. Thus, there was more room for improvements in blood lipid levels, adiposity, and CRF than there was for glycemic metabolism. Despite their weight status, our participants were at healthy (low) glycemic and blood pressure levels at baseline, which could produce a floor effect.

Children in the exercise group improved their body composition by reducing their total and visceral fat mass. These results are in line with the previous literature studying children with obesity regarding the reductions in body mass index and fat mass.^11,12,13,14^ Likewise, a recent network meta-analysis^64^ concluded that aerobic or the combined aerobic and resistance training effectively reduced adiposity outcomes with similar magnitude as we observed in our study (body mass index of approximately 0.7 vs 0.59 in our study) in children and adolescents with overweight or obesity. No less important, we found that a higher rate of participants experienced clinically relevant change (ie, at least 5% reduction) in their fat mass index, which is in line with the EFIGRO (Effect of Exercise on Hepatic Fat in Overweight Children) trial findings.^22^ Our lean mass index was not affected by the exercise program, which agrees with a previous study using a similar indicator of lean mass.^11^ However, another RCT in children with obesity with a similar dose of resistance training^14^ described improvements in fat-free mass (+1.2 kg compared with controls).

Regarding physical fitness, the exercise program improved CRF, both the performance in the test (laps) and the estimated V̇o_2_max. These results agree with previous trials in children with overweight or obesity.^11,12,13,14^ The exercise program did not improve the children’s speed-agility or muscular fitness, a finding consistent with those from the EFIGRO trial.^11^ The specificity of our resistance exercises might explain this null finding—that is, body-weight exercises instead of weightlifting may have produced benefits in muscular endurance instead of maximal strength or power (as measured by the handgrip and the standing long-jump tests).

None of the previous studies have analyzed the effects of exercise programs on composite cardiometabolic risk scores, which hampers comparisons in this regard. We believe this is a strength of our study to quantify the effect on the composite cardiometabolic risk scores, which are valid measures of risk for type 2 diabetes, CVD, and other cardiometabolic diseases,^38^ and a better marker of cardiovascular health in children than using a single risk factor.^18^ Our findings are further strengthened by the investigation of the proportion of children experiencing meaningful changes in the control and the exercise groups.

### Mental Health

The exercise program did not improve mental health in children with overweight or obesity. The null findings for mental health might be due to the ceiling effect—that is, most of the children had a healthy mental status at baseline. Indeed, consistent exercise effects have been observed on depression in adolescents.^19,66,67^ This is likely explained because children are still young and present high levels of well-being and low levels of ill-being, which makes it unnecessary and complicated to improve these outcomes further. The effects of exercise on mental health in children and adolescents are inconsistent^67^ and differ according to a range of contextual factors (eg, type of activity, delivery mode) and participant characteristics (eg, age, clinical diagnosis).^68^ Seabra et al^69^ concluded that a 20-week football program improved self-esteem in boys with overweight. Alternatively, Romero-Perez et al^70^ found no significant changes in anxiety and a small reduction in depression in children with obesity after 20 weeks of aerobic exercise. Williams et al^15^ observed that an 8-month aerobic exercise after-school program provided benefits to quality of life, depressive symptoms, and self-worth in children with overweight. Differences in our findings and the previous studies could be explained by the heterogeneity of the exercise program (type: only aerobic vs aerobic and resistance training; frequency: 2 vs 3 to 5 sessions per week), characteristics of the study sample (sex, weight status), the mental health outcomes examined (individual dimensions vs a complete set of psychological ill-being and well-being outcomes); and the study design (non-RCT vs RCT).

Although our intervention complied with most of the SAAFE principles (ie, Supportive, Active, Autonomous, Fair, and Enjoyable)^71^ to maximize the effects of exercise on mental health, we did not assess the session fidelity. Therefore, we cannot confirm that the sessions adhered to these principles. Alternatively, the lack of sensitivity of our mental health measures and/or the ceiling effect experienced in our children may explain the null findings.

### Limitations

Our findings might be limited by the relatively small sample size (nonrepresentative), which could make some of the statistical analyses underpowered to detect significant differences and by the fact that some of the evaluators were not blinded to the group allocation. We believe that most of the outcomes included in our study are objective and unlikely to be influenced by assessor blinding (ie, cardiometabolic health, blood markers assessed in external laboratory, and body composition by dual-energy x-ray absorptiometry). The lack of findings in mental health may be explained by the ceiling effect observed in our children (ie, healthy mental status at baseline).

## Conclusion

In this secondary analysis of the ActiveBrains RCT, the current aerobic plus resistance exercise program improved cardiometabolic health in children with overweight or obesity. The cardiometabolic risk score was reduced by approximately 0.38 SDs, which was mainly due to the improvements observed in blood lipid levels, total and visceral adiposity, and CRF. However, our intervention did not affect any of the mental health studied. These findings demonstrate the potential of exercise programs to promote cardiometabolic health in children with overweight and obesity, which may have implications for public health. However, further studies are needed to examine a larger-scale and longer public health intervention combining exercise programs with the promotion of other important health behaviors (eg, healthy diet). The null effect on mental health outcomes needs to be further investigated, including, among other things, whether the instruments are sensitive enough to detect changes and whether there is a ceiling effect in young children who might be mentally healthy overall.

## Notes

Supplement 1.
Trial Protocol

Supplement 2. **eTable 1.** Descriptive Characteristics of the ActiveBrains Participants Meeting the Per-Protocol Criteria at Baseline **eTable 2.** Descriptive Characteristics of the ActiveBrains Adherent and Nonadherent Participants at Baseline **eTable 3.** Effects of the ActiveBrains Exercise Program on Cardiometabolic Health (Per-Protocol Analyses) **eTable 4.** Effects of the ActiveBrains Exercise Program on Cardiometabolic Health (Intention-to-Treat Analyses) **eTable 5.** Effects of the ActiveBrains Exercise Program on Mental Health (Per-Protocol Analyses) **eTable 6.** Effects of the ActiveBrains Exercise Program on Mental Health (Intention-to-Treat Analyses) **eFigure 1.** Individual Change Distribution in the Cardiometabolic Risk Scores, Visceral Adipose Tissue, and Body Mass Index Affected by the Exercise Program **eFigure 2.** Change in Daily Activity Composition Induced by the Exercise Program as Measured With Hip- and Wrist-Worn Accelerometers

Supplement 3.
Data Sharing Statement
