# Error in Supplement

**DOI:** 10.1001/jamanetworkopen.2024.19953

**Published:** 2024-05-31

In the Original Investigation titled “Effects of an Exercise Program on Cardiometabolic and Mental Health in Children With Overweight or Obesity: A Secondary Analysis of a Randomized Clinical Trial,”^1^ published July 27, 2023, many of the SDs given in eTable 1 in Supplement 2 were incorrect, as they were duplicates of their corresponding means. This article has been corrected.
