# Supplementary material for: Effects of an Exercise Program on Cardiometabolic and Mental Health in Children With Overweight or Obesity: A Secondary Analysis of a Randomized Clinical Trial
Source: JAMA Netw Open. 2023 Jul 27;6(7):e2324839. doi: 10.1001/jamanetworkopen.2023.24839 (PMC10375312; doi:10.1001/jamanetworkopen.2023.24839)
Supplement: Supplement 2. — eTable 1. Descriptive Characteristics of the ActiveBrains Participants Meeting the Per-Protocol Criteria at Baseline eTable 2. Descriptive Characteristics of the ActiveBrains Adherent and Nonadherent Participants at Baseline eTable 3. Effects of the ActiveBrains Exercise Program on Cardiometabolic Health (Per-Protocol Analyses) eTable 4. Effects of the ActiveBrains Exercise Program on Cardiometabolic Health (Intention-to-Treat Analyses) eTable 5. Effects of the ActiveBrains Exercise Program on Mental Health (Per-Protocol Analyses) eTable 6. Effects of the ActiveBrains Exercise Program on Mental Health (Intention-to-Treat Analyses) eFigure 1. Individual Change Distribution in the Cardiometabolic Risk Scores, Visceral Adipose Tissue, and Body Mass Index Affected by the Exercise Program eFigure 2. Change in Daily Activity Composition Induced by the Exercise Program as Measured With Hip- and Wrist-Worn Accelerometers [file jamanetwopen-e2324839-s002.pdf]

Supplementary Online Content

Migueles JH, Cadenas-Sanchez C, Lubans DR, et al. Effects of an exercise program on cardiometabolic and mental health in children with overweight or obesity: a secondary analysis of a randomized clinical trial. *JAMA Netw Open*. 2023;6(7):e2324839. doi:10.1001/jamanetworkopen.2023.24839

eTable 1. Descriptive Characteristics of the ActiveBrains Participants Meeting the Per-Protocol Criteria at Baseline

eTable 2. Descriptive Characteristics of the ActiveBrains Adherent and Nonadherent Participants at Baseline

eTable 3. Effects of the ActiveBrains Exercise Program on Cardiometabolic Health (Per-Protocol Analyses)

eTable 4. Effects of the ActiveBrains Exercise Program on Cardiometabolic Health (Intention-to-Treat Analyses)

eTable 5. Effects of the ActiveBrains Exercise Program on Mental Health (Per-Protocol Analyses)

eTable 6. Effects of the ActiveBrains Exercise Program on Mental Health (Intention-to-Treat Analyses)

eFigure 1. Individual Change Distribution in the Cardiometabolic Risk Scores, Visceral Adipose Tissue, and Body Mass Index Affected by the Exercise Program

eFigure 2. Change in Daily Activity Composition Induced by the Exercise Program as Measured With Hip- and Wrist-Worn Accelerometers

This supplementary material has been provided by the authors to give readers additional information about their work.

© 2023 Migueles JH et al. *JAMA Network Open*.

eTable 1. Descriptive characteristics of the ActiveBrains participants meeting the per-protocol criteria at baseline.

	All		Control group		Exercise group	
	N	Mean (SD) or %	N	Mean (SD) or %	N	Mean (SD) or %
Sex						
Girls (n %)	41	41%	25	48%	16	39%
Boys (n %)	58	59%	27	52%	31	76%
At risk of dyslipidemia (n %)	43	44%	27	52%	16	28%
Pre-diabetes (n %)	3	3%	0	0%	3	6%
Pre-hypertension (n %)	10	10%	5	10%	5	9%
Unfit (n %)	62	63%	32	62%	30	53%
At risk of metabolic syndrome (n %)	25	25%	7	13%	18	38%
At risk of anxiety (n %)	18	19%	11	22%	7	16%
At risk of depression (n %)	3	3%	1	2%	2	4%
Age (y)	99	10.0 (1.1)	51	10.1 (1.1)	47	10.0 (1.1)
Peak height velocity (y)	99	-2.2 (1.0)	51	-2.1 (1.1)	47	-2.4 (0.9)
Cardiometabolic health						
LDL (mg/dL)	86	100.7 (24.63)	45	102.4 (24.55)	41	98.9 (24.90)
HDL (mg/dL)	95	51.0 (11.05)	50	49.7 (10.54)	45	52.4 (11.54)
Triglycerides (mg/dL)	95	95.8 (46.09)	50	99.0 (48.86)	45	92.2 (43.06)
Triglycerides-to-HDL (mg/dL)	95	2.1 (1.63)	50	2.2 (1.53)	45	2.0 (1.75)
Insulin (mg/dL)	90	13.5 (8.72)	47	13.2 (8.67)	43	13.7 (8.86)
Glucose (mg/dL)	96	86.3 (6.90)	50	84.7 (6.53)	46	88.0 (6.97)
HOMA	89	2.9 (1.99)	46	2.8 (1.94)	43	3.0 (2.05)
Systolic blood pressure (mmHG)	97	100.5 (13.66)	50	100.1 (14.10)	47	101.1 (13.31)
Diastolic blood pressure (mmHG)	97	57.2 (13.66)	50	56.5 (14.38)	47	58.0 (12.97)
Body mass index (kg/m^2)	99	26.8 (3.56)	52	26.3 (2.97)	47	27.4 (4.07)
Body mass index (WHO z-score)	99	3.0 (0.87)	52	2.9 (0.88)	47	3.1 (0.87)
Fat mass index (kg/m^2)	98	11.8 (2.82)	51	11.3 (2.28)	47	12.4 (3.24)
Lean mass index (kg/m^2)	98	13.9 (1.48)	51	13.9 (1.40)	47	14.0 (1.57)
Waist circumference (cm)	99	90.5 (9.77)	52	89.7 (8.50)	47	91.3 (11.03)
Visceral adipose tissue (g)	80	402.1 (114.15)	42	390.4 (104.61)	38	415.1 (123.95)
CRF performance (laps)	97	15.6 (7.12)	50	16.1 (7.38)	47	15.0 (6.86)
CRF (VO_2max, ml/kg/min)	97	40.6 (2.65)	50	40.7 (2.33)	47	40.4 (2.98)
Speed-agility fitness (s)	97	15.1 (1.57)	50	15.0 (1.66)	47	15.3 (1.47)
Upper-limb muscular strength (kg)	98	16.9 (4.25)	51	17.1 (4.05)	47	16.7 (4.49)
Lower-limb muscular strength (cm)	97	104.4 (18.10)	50	106.3 (19.04)	47	102.3 (17.00)
Children-reported mental health						
Stress (0 – 30)*	96	5.8 (3.29)	50	6.2 (3.16)	46	5.4 (3.41)
Anxiety (20 – 60)*	95	33.6 (7.29)	50	34.1 (7.10)	45	33.0 (7.54)
Depression (0 – 54)*	96	8.4 (5.12)	50	9.0 (5.31)	46	7.8 (4.88)
Negative affect (10 – 30)*	95	16.0 (3.51)	50	16.3 (3.58)	45	15.7 (3.46)
Positive affect (10 – 30)*	97	24.4 (2.97)	51	24.5 (2.97)	46	24.3 (2.99)
Happiness (4 – 28)*	99	22.7 (3.92)	52	22.4 (3.72)	47	23.1 (4.14)
Optimism (6 – 30)*	98	22.0 (4.04)	51	22.1 (4.23)	47	22.0 (3.88)
Self-efficacy (10 – 40)*	98	30.9 (4.92)	51	30.5 (5.36)	47	31.3 (4.41)
Self-concept (30 – 300)*	97	227.1 (28.88)	50	225.4 (29.86)	47	228.8 (28.02)
Self-esteem (10 – 40)*	98	33.0 (4.69)	51	32.6 (4.51)	47	33.3 (4.91)

Data analyses were primarily conducted under the per-protocol principle, i.e., attending 70% of the sessions or keep the usual lifestyle for exercise and control groups, respectively.
*Score range for the questionnaire.
LDL: low-density lipoprotein; HDL: high-density lipoprotein; HOMA: homeostatic model assessment; CRF: cardiorespiratory fitness; VO_2max: maximum oxygen consumption, WHO: World Health Organization.

© 2023 Migueles JH et al. *JAMA Network Open*.

eTable 2. Descriptive Characteristics of the ActiveBrains Adherent and Nonadherent Participants at Baseline

	Included		Dropouts		
	N	Mean (SD) or %	N	Mean (SD) or %	*P*
Sex					0.427
Girls (n %)	36	39%	9	53%	
Boys (n %)	56	61%	8	47%	
Maternal education level					0.627
University degree (n %)	24	26%	6	35%	
Primary or secondary school (n %)	68	74%	11	65%	
Age (y)	92	10.0 (1.1)	17	10.1 (1.2)	0.871
Peak height velocity (y)	92	-2.3 (1.0)	17	-2.2 (1.0)	0.635
Body mass index (kg/m^2)	92	26.7 (3.6)	17	27.3 (3.7)	0.523
Body mass index (WHO z-score)	92	3.0 (0.9)	17	3.1 (0.9	0.857
Fat mass index (kg/m^2)	92	11.7 (2.8)	17	12.5 (3.6)	0.397

© 2023 Migueles JH et al. *JAMA Network Open*.

eTable 3. Effects of the ActiveBrains Exercise Program on Cardiometabolic Health (Per-Protocol Analyses)

	Mean (95% CI)						
	N_{all}	N	Exercise group*	N	Control group*	Difference between groups	*P*
LDL cholesterol (mg/dL)	71	38		33			
Raw score			90.35 (85.37 to 95.33)		97.3 (91.95 to 102.64)	-6.95 (-14.27 to 0.37)	0.06
Z-Score			-0.42 (-0.62 to -0.22)		-0.14 (-0.36 to 0.08)	-0.28 (-0.58 to 0.02)	
HDL cholesterol (mg/dL)†	79	42		37			
Raw score			51.57 (48.99 to 54.15)		49.08 (46.33 to 51.83)	2.49 (-1.3 to 6.27)	0.19
Z-Score			0.04 (-0.2 to 0.27)		-0.19 (-0.44 to 0.06)	0.23 (-0.12 to 0.57)	
Triglycerides (mg/dL)	86	44		42			
Raw score			94.15 (83.97 to 104.33)		97.63 (87.21 to 108.05)	-3.48 (-18.06 to 11.09)	0.63
Z-Score			-0.05 (-0.27 to 0.17)		0.03 (-0.2 to 0.26)	-0.08 (-0.39 to 0.24)	
TG-to-HDL ratio (mg/dL)	78	41		37			
Raw score			1.88 (1.59 to 2.17)		2.12 (1.82 to 2.42)	-0.24 (-0.65 to 0.18)	0.26
Z-Score			-0.16 (-0.33 to 0.02)		-0.01 (-0.2 to 0.17)	-0.14 (-0.4 to 0.11)	
Insulin (µU/ml)	76	39		37			
Raw score			13.36 (11.31 to 15.41)		13.91 (11.8 to 16.02)	-0.55 (-3.49 to 2.39)	0.71
Z-Score			-0.01 (-0.25 to 0.22)		0.05 (-0.19 to 0.29)	-0.06 (-0.4 to 0.27)	
Glucose (mg/dL)	88	46		42			
Raw score			83.98 (81.59 to 86.38)		83.76 (81.24 to 86.27)	0.23 (-3.29 to 3.75)	0.89
Z-Score			-0.33 (-0.67 to 0.02)		-0.36 (-0.72 to 0)	0.03 (-0.47 to 0.54)	
HOMA	75	39		36			
Raw score			2.81 (2.3 to 3.32)		2.93 (2.4 to 3.46)	-0.12 (-0.86 to 0.61)	0.74
Z-Score			-0.04 (-0.3 to 0.22)		0.02 (-0.25 to 0.29)	-0.06 (-0.43 to 0.31)	
Systolic and diastolic average (mmHG)	87	46		41			
Raw score			78.01 (75.76 to 80.26)		79.88 (77.49 to 82.26)	-1.86 (-5.14 to 1.42)	0.26
Z-Score			-0.05 (-0.23 to 0.14)		0.11 (-0.09 to 0.3)	-0.15 (-0.42 to 0.12)	
MAP (mmHG)	87	46		41			
Raw score			70.1 (67.93 to 72.27)		71.32 (69.02 to 73.62)	-1.22 (-4.38 to 1.95)	0.44
Z-Score			-0.1 (-0.28 to 0.08)		0 (-0.19 to 0.19)	-0.1 (-0.36 to 0.16)	
Body mass index (kg/m^2)	92	47		45			
Raw score			26.34 (26.02 to 26.67)		26.93 (26.59 to 27.26)	-0.59 (-1.06 to -0.12)	0.01
Z-Score			-0.13 (-0.23 to -0.04)		0.03 (-0.06 to 0.12)	-0.16 (-0.3 to -0.03)	
WHO z-Score			-0.69 (-0.75 to -0.63)		-0.59 (-0.65 to -0.53)	-0.1 (-0.19 to -0.01)	0.02

© 2023 Migueles JH et al. *JAMA Network Open*.

Fat mass index (kg/m^2)	92	47		45			
Raw score			10.75 (10.52 to 10.99)		11.42 (11.18 to 11.66)	-0.67 (-1.01 to -0.33)	0.001
Z-Score			-0.37 (-0.46 to -0.28)		-0.1 (-0.2 to -0.01)	-0.27 (-0.4 to -0.14)	
Lean mass index (kg/m^2)	92	47		45			
Raw score			13.58 (13.38 to 13.79)		13.54 (13.33 to 13.75)	0.05 (-0.25 to 0.34)	0.75
Z-Score			-0.24 (-0.38 to -0.1)		-0.27 (-0.41 to -0.13)	0.03 (-0.17 to 0.23)	
Waist circumference (cm)	92	47		45			
Raw score			91.85 (90.75 to 92.95)		93.02 (91.89 to 94.14)	-1.17 (-2.74 to 0.41)	0.14
Z-Score			0.14 (0.03 to 0.25)		0.26 (0.14 to 0.37)	-0.12 (-0.28 to 0.04)	
Visceral adipose tissue (g)	77	38		39			
Raw score			394.66 (375.13 to 414.19)		426.1 (406.83 to 445.38)	-31.44 (-58.99 to -3.9)	0.02
Z-Score			-0.07 (-0.24 to 0.11)		0.21 (0.04 to 0.38)	-0.28 (-0.52 to -0.03)	
CRF performance (laps)	89	46		43			
Raw score			18.85 (17.1 to 20.6)		16.09 (14.28 to 17.9)	2.75 (0.22 to 5.28)	0.03
Z-Score			0.46 (0.22 to 0.71)		0.08 (-0.18 to 0.33)	0.39 (0.03 to 0.74)	
CRF VO_2max (ml/kg/min)	89	46		43			
Raw score			40.78 (40.16 to 41.4)		39.84 (39.2 to 40.48)	0.94 (0.05 to 1.84)	0.03
Z-Score			0.08 (-0.16 to 0.31)		-0.28 (-0.52 to -0.03)	0.35 (0.02 to 0.69)	
Speed-agility fitness (sec)‡	89	46		43			
Raw score			14.84 (14.59 to 15.08)		14.96 (14.71 to 15.21)	-0.13 (-0.48 to 0.22)	0.46
Z-Score			-0.18 (-0.33 to -0.02)		-0.1 (-0.26 to 0.06)	-0.08 (-0.3 to 0.14)	
Upper-limb muscular strength (kg)	90	47		43			
Raw score			17.45 (16.93 to 17.97)		17.98 (17.43 to 18.53)	-0.53 (-1.29 to 0.23)	0.17
Z-Score			0.13 (0.01 to 0.26)		0.26 (0.13 to 0.39)	-0.12 (-0.3 to 0.05)	
Lower-limb muscular strength (cm)	90	47		43			
Raw score			106.45 (103.14 to 109.77)		109.27 (105.81 to 112.74)	-2.82 (-7.64 to 2)	0.24
Z-Score			0.11 (-0.07 to 0.3)		0.27 (0.08 to 0.46)	-0.16 (-0.42 to 0.11)	
Cardiometabolic risk score 1**	75	41	-0.163 (-0.405 to 0.079)	43	0.196 (-0.069 to 0.462)	-0.359 (-0.719 to 0.001)	0.05
Cardiometabolic risk score 2††	75	41	-0.172 (-0.412 to 0.068)	43	0.208 (-0.056 to 0.472)	-0.38 (-0.738 to -0.021)	0.03

Data analyses were primarily conducted under the per-protocol principle, i.e., attending 70% of the sessions.
Z-Score values indicate how many standard deviations have the follow-up values changed with respect to the baseline mean and standard deviation. E.g., a 0.50 Z-Score means that the mean value at follow-up is 0.50 standard deviations higher than the mean value at baseline indicating a positive change with negative values indicating the opposite.
*Adjusted for baseline values.

© 2023 Migueles JH et al. *JAMA Network Open*.

†Higher values indicate better health.
‡Higher values indicate lower performance.
**Cardiometabolic risk score 1 was calculated as the age- and sex-normalized scores for HDL cholesterol, waist circumference, triglycerides, glucose, and the average of systolic and diastolic blood pressure based on European population reference values.
††Cardiometabolic risk score 2 additionally included the CRF.
TG: triglyceride; LDL: low density lipoprotein; HDL: high lipoprotein consumption; MAP: mean arterial pressure; CRF: cardiorespiratory fitness; VO_2max: maximum oxygen consumption.

© 2023 Migueles JH et al. *JAMA Network Open*.

eTable 4. Effects of the ActiveBrains Exercise Program on Cardiometabolic Health (Intention-to-Treat Analyses)

	Mean (95% CI)						
	N_{all}	N	Exercise group*	N	Control group*	Difference between groups	*P*
LDL cholesterol (mg/dL)	109	57		52			
Raw score			92.914 (88.739 to 97.089)		94.69 (90.319 to 99.062)	-1.776 (-7.829 to 4.276)	0.56
Z-Score			-0.32 (-0.487 to -0.153)		-0.249 (-0.424 to -0.074)	-0.071 (-0.313 to 0.171)	
HDL cholesterol (mg/dl)†	109	57		52			
Raw score			51.981 (49.862 to 54.1)		49.198 (46.979 to 51.417)	2.783 (-0.291 to 5.857)	0.03
Z-Score			0.132 (-0.066 to 0.33)		-0.128 (-0.335 to 0.079)	0.26 (-0.027 to 0.547)	
Triglycerides (mg/dl)	109	57		52			
Raw score			94.085 (85.467 to 102.703)		97.81 (88.786 to 106.835)	-3.725 (-16.225 to 8.775)	0.55
Z-Score			-0.043 (-0.234 to 0.149)		0.04 (-0.161 to 0.24)	-0.083 (-0.361 to 0.195)	
TG-to-HDL ratio (mg/dl)	109	57		52			
Raw score			1.934 (1.707 to 2.16)		2.124 (1.887 to 2.361)	-0.19 (-0.519 to 0.138)	0.25
Z-Score			-0.122 (-0.267 to 0.023)		0 (-0.152 to 0.151)	-0.122 (-0.332 to 0.088)	
Insulin (µU/ml)	109	57		52			
Raw score			13.147 (11.681 to 14.614)		13.669 (12.133 to 15.204)	-0.522 (-2.645 to 1.602)	0.62
Z-Score			-0.037 (-0.217 to 0.142)		0.026 (-0.161 to 0.214)	-0.064 (-0.323 to 0.196)	
Glucose (mg/dl)	109	57		52			
Raw score			84.597 (82.539 to 86.656)		84.041 (81.883 to 86.199)	0.556 (-2.469 to 3.582)	0.71
Z-Score			-0.283 (-0.592 to 0.025)		-0.367 (-0.69 to -0.043)	0.083 (-0.37 to 0.537)	
HOMA	109	57		52			
Raw score			2.803 (2.442 to 3.164)		2.894 (2.516 to 3.272)	-0.091 (-0.614 to 0.431)	0.72
Z-Score			-0.048 (-0.243 to 0.147)		0.002 (-0.203 to 0.206)	-0.049 (-0.332 to 0.233)	
Systolic and diastolic average (mmHG)	109	57		52			
Raw score			78.229 (76.37 to 80.089)		79.859 (77.912 to 81.807)	-1.63 (-4.326 to 1.066)	0.23
Z-Score			-0.084 (-0.236 to 0.067)		0.048 (-0.11 to 0.207)	-0.133 (-0.352 to 0.087)	
MAP (mmHG)	109	57		52			
Raw score			70.356 (68.535 to 72.177)		71.521 (69.614 to 73.428)	-1.164 (-3.804 to 1.476)	0.38
Z-Score			-0.132 (-0.277 to 0.013)		-0.039 (-0.191 to 0.112)	-0.093 (-0.302 to 0.117)	
Body mass index (kg/m^2)	109	57		52			
Raw score			26.585 (26.27 to 26.899)		26.903 (26.574 to 27.232)	-0.319 (-0.776 to 0.139)	0.17
Z-Score			-0.062 (-0.149 to 0.025)		0.026 (-0.065 to 0.117)	-0.088 (-0.214 to 0.038)	
WHO z-Score			-0.605 (-0.665 to -0.545)		-0.556 (-0.618 to -0.493)	-0.049 (-0.136 to 0.037)	0.26
Fat mass index (kg/m^2)	109	57		52			

© 2023 Migueles JH et al. *JAMA Network Open*.

Raw score			13.578 (13.375 to 13.782)		13.66 (13.447 to 13.873)	-0.082 (-0.376 to 0.213)	0.01
Z-Score			-0.274 (-0.414 to -0.134)		-0.218 (-0.365 to -0.071)	-0.056 (-0.259 to 0.147)	
Lean mass index (kg/m^2)	109	57		52			
Raw score			92.124 (91.128 to 93.119)		92.78 (91.738 to 93.822)	-0.656 (-2.098 to 0.785)	0.58
Z-Score			0.194 (0.094 to 0.295)		0.261 (0.155 to 0.366)	-0.066 (-0.212 to 0.079)	
Waist circumference (cm)	109	57		52			
Raw score			397.313 (381.94 to 412.686)		417.309 (401.213 to 433.406)	-19.996 (-42.274 to 2.282)	0.36
Z-Score			-0.02 (-0.168 to 0.128)		0.172 (0.017 to 0.327)	-0.193 (-0.407 to 0.022)	
Visceral adipose tissue (g)	109	57		52			
Raw score			19.269 (17.434 to 21.103)		17.928 (16.008 to 19.849)	1.34 (-1.315 to 3.996)	0.07
Z-Score			0.424 (0.185 to 0.662)		0.249 (-0.001 to 0.499)	0.174 (-0.171 to 0.52)	
CRF performance (laps)	109	57		52			
Raw score			40.943 (40.34 to 41.545)		40.277 (39.646 to 40.908)	0.666 (-0.206 to 1.539)	0.31
Z-Score			0.086 (-0.137 to 0.309)		-0.161 (-0.394 to 0.073)	0.247 (-0.076 to 0.57)	
CRF VO_2max (ml/kg/min)	109	57		52			
Raw score			14.787 (14.552 to 15.023)		14.849 (14.602 to 15.095)	-0.061 (-0.403 to 0.28)	0.13
Z-Score			-0.207 (-0.357 to -0.057)		-0.168 (-0.325 to -0.01)	-0.039 (-0.257 to 0.179)	
Speed-agility (sec)‡	109	57		52			
Raw score			17.641 (17.155 to 18.127)		18.026 (17.517 to 18.535)	-0.384 (-1.089 to 0.32)	0.72
Z-Score			0.193 (0.076 to 0.31)		0.286 (0.163 to 0.408)	-0.093 (-0.262 to 0.077)	
Upper-limb muscular strength (kg)	109	57		52			
Raw score			107.434 (104.459 to 110.41)		108.789 (105.673 to 111.905)	-1.355 (-5.669 to 2.959)	0.28
Z-Score			0.129 (-0.034 to 0.292)		0.204 (0.033 to 0.374)	-0.074 (-0.311 to 0.162)	
Lower-limb muscular strength (cm)	109	57		52			
Raw score			-0.118 (-0.324 to 0.089)		0.129 (-0.087 to 0.345)	-0.247 (-0.546 to 0.053)	0.53
Z-Score			-0.121 (-0.325 to 0.083)		0.133 (-0.081 to 0.346)	-0.254 (-0.55 to 0.042)	
Cardiometabolic risk score 1**	109	57	13.578 (13.375 to 13.782)	52	13.66 (13.447 to 13.873)	-0.082 (-0.376 to 0.213)	0.10
Cardiometabolic risk score 2††	109	57	-0.274 (-0.414 to -0.134)	52	-0.218 (-0.365 to -0.071)	-0.056 (-0.259 to 0.147)	0.09

Data analyses were conducted under the intention-to-treat principle, i.e., including all participants and imputing the missing data using predictive mean matching multiple imputations. Z-score values indicate how many standard deviations have the follow-up values changed with respect to the baseline mean and standard deviation. E.g., a 0.50 Z-score means that the mean value at follow-up is 0.50 standard deviations higher than the mean value at baseline indicating a positive change with negative values indicating the opposite.

*Adjusted for baseline values.

© 2023 Migueles JH et al. *JAMA Network Open*.

[†]Higher values indicate better health.
[‡]Higher values indicate lower performance
**Cardiometabolic risk score 1 was calculated as the age- and sex-normalized scores for HDL cholesterol, waist circumference, triglycerides, glucose, and the average of systolic and diastolic blood pressure based on European population reference values.
[††]Cardiometabolic risk score 2 additionally included the CRF.
TG: triglyceride; LDL: low density lipoprotein; HDL: high lipoprotein consumption; MAP: mean arterial pressure; CRF: cardiorespiratory fitness; VO_2max: maximum oxygen consumption.

© 2023 Migueles JH et al. *JAMA Network Open*.

eTable 5. Effects of the ActiveBrains Exercise Program on Mental Health (Per-Protocol Analyses)

	Mean (95% CI)						
	N_{all}	N	Exercise group*	N	Control group*	Difference between groups	*P*
Psychological ill-being†							
Stress	88	46		42			
Raw score			5.17 (4.38 to 5.96)		5.05 (4.22 to 5.88)	0.12 (-1.04 to 1.27)	0.84
Z-Score			-0.2 (-0.44 to 0.04)		-0.24 (-0.49 to 0.02)	0.04 (-0.32 to 0.39)	
Anxiety	84	45		39			
Raw score			32.04 (30.16 to 33.92)		30.21 (28.19 to 32.23)	1.83 (-0.93 to 4.59)	0.19
Z-Score			-0.21 (-0.47 to 0.05)		-0.46 (-0.74 to -0.18)	0.25 (-0.13 to 0.63)	
Depression	88	44		44			
Raw score			7.31 (5.91 to 8.7)		7.31 (5.91 to 8.7)	0 (-1.98 to 1.98)	0.99
Z-Score			-0.21 (-0.49 to 0.06)		-0.21 (-0.49 to 0.06)	0 (-0.39 to 0.39)	
Negative affect	80	44		36			
Raw score			15.71 (14.67 to 16.74)		16.16 (15.02 to 17.31)	-0.45 (-2 to 1.1)	0.56
Z-Score			-0.09 (-0.39 to 0.2)		0.04 (-0.29 to 0.36)	-0.13 (-0.57 to 0.31)	
Composite score	73	39	0.07 (-0.13 to 0.27)	34	-0.07 (-0.28 to 0.15)	0.14 (-0.15 to 0.43)	0.34
Psychological well-being							
Positive affect	81	44		37			
Raw score			24.93 (23.98 to 25.88)		24.94 (23.91 to 25.98)	-0.01 (-1.42 to 1.39)	0.95
Z-Score			0.18 (-0.14 to 0.5)		0.18 (-0.17 to 0.53)	0 (-0.48 to 0.47)	
Happiness	92	47		45			
Raw score			22.79 (21.78 to 23.8)		23.6 (22.56 to 24.63)	-0.81 (-2.26 to 0.64)	0.30
Z-Score			0.01 (-0.24 to 0.27)		0.22 (-0.04 to 0.48)	-0.21 (-0.58 to 0.16)	
Optimism	87	46		41			
Raw score			22.56 (21.44 to 23.68)		22.32 (21.14 to 23.51)	0.24 (-1.39 to 1.86)	0.64
Z-Score			0.13 (-0.15 to 0.41)		0.07 (-0.22 to 0.37)	0.06 (-0.34 to 0.46)	
Self-efficacy	90	47		43			
Raw score			31.05 (29.7 to 32.41)		31.61 (30.2 to 33.03)	-0.56 (-2.52 to 1.4)	0.65
Z-Score			0.04 (-0.23 to 0.32)		0.15 (-0.13 to 0.44)	-0.11 (-0.51 to 0.29)	
Self-concept	79	43		36			
Raw score			219.53 (211.27 to 227.78)		223.15 (214.12 to 232.17)	-3.62 (-15.86 to 8.62)	0.55
Z-Score			-0.26 (-0.55 to 0.02)		-0.14 (-0.45 to 0.18)	-0.13 (-0.55 to 0.3)	
Self-esteem	87	47		40			
Raw score			35.17 (33.99 to 36.35)		34.8 (33.52 to 36.08)	0.37 (-1.37 to 2.11)	0.60

© 2023 Migueles JH et al. *JAMA Network Open*.

Z-Score			0.47 (0.22 to 0.72)		0.39 (0.12 to 0.66)	0.08 (-0.29 to 0.45)	
Composite score	72	41	-0.05 (-0.3 to 0.2)	31	0.08 (-0.21 to 0.36)	-0.13 (-0.51 to 0.25)	0.50
Total mental health							
Composite score	64	36	-0.12 (-0.36 to 0.12)	28	0.11 (-0.16 to 0.39)	-0.24 (-0.6 to 0.13)	0.20

Data analyses were primarily conducted under the per-protocol principle, i.e., attending 70% of the sessions. Z-score values indicate how many standard deviations have the follow-up values changed with respect to the baseline mean and standard deviation. E.g., a 0.50 Z-score means that the mean value at follow-up is 0.50 standard deviations higher than the mean value at baseline, indicating a positive change with negative values indicating the opposite.

*Adjusted for baseline values.

†Higher values indicate lower mental health.

© 2023 Migueles JH et al. *JAMA Network Open*.

eTable 6. Effects of the ActiveBrains Exercise Program on Mental Health (Intention-to-Treat Analyses)

	Mean (95% CI)						
	N_{all}	N	Exercise group*	N	Control group*	Difference between groups	P
Psychological ill-being†							
Stress	109	57		52			
Raw score			5.24 (4.49 to 5.98)		5.13 (4.34 to 5.92)	0.11 (-0.98 to 1.20)	0.84
Z-Score			-0.16 (-0.4 to 0.08)		-0.19 (-0.45 to 0.06)	0.03 (-0.32 to 0.39)	
Anxiety	109	57		52			
Raw score			32.11 (30.53 to 33.68)		30.94 (29.27 to 32.61)	1.17 (-1.13 to 3.46)	0.31
Z-Score			-0.2 (-0.41 to 0.02)		-0.36 (-0.59 to -0.13)	0.16 (-0.16 to 0.48)	
Depression	109	57		52			
Raw score			7.42 (6.19 to 8.66)		7.8 (6.49 to 9.1)	-0.37 (-2.18 to 1.43)	0.68
Z-Score			-0.19 (-0.42 to 0.05)		-0.12 (-0.37 to 0.13)	-0.07 (-0.41 to 0.27)	
Negative affect	109	57		52			
Raw score			15.72 (14.87 to 16.58)		16.33 (15.42 to 17.23)	-0.61 (-1.85 to 0.64)	0.33
Z-Score			-0.14 (-0.39 to 0.12)		0.04 (-0.23 to 0.31)	-0.18 (-0.55 to 0.19)	
Composite score	109	57	-0.02 (-0.22 to 0.18)	52	0.02 (-0.19 to 0.23)	-0.03 (-0.33 to 0.26)	0.81
Psychological well-being							
Positive affect	109	57		52			
Raw score			24.71 (23.83 to 25.59)		24.7 (23.77 to 25.63)	0.01 (-1.27 to 1.29)	0.98
Z-Score			0.09 (-0.21 to 0.38)		0.08 (-0.23 to 0.39)	0 (-0.42 to 0.43)	
Happiness	109	57		52			
Raw score			23.01 (22.1 to 23.92)		23.1 (22.14 to 24.06)	-0.09 (-1.42 to 1.24)	0.89
Z-Score			0.07 (-0.16 to 0.29)		0.09 (-0.15 to 0.33)	-0.02 (-0.36 to 0.31)	
Optimism	109	57		52			
Raw score			22.73 (21.73 to 23.74)		21.9 (20.83 to 22.97)	0.83 (-0.63 to 2.30)	0.26
Z-Score			0.21 (-0.05 to 0.46)		-0.01 (-0.28 to 0.27)	0.21 (-0.16 to 0.58)	
Self-efficacy	109	57		52			
Raw score			31.47 (30.29 to 32.64)		31.62 (30.38 to 32.87)	-0.16 (-1.88 to 1.56)	0.85
Z-Score			0.11 (-0.13 to 0.36)		0.15 (-0.11 to 0.41)	-0.03 (-0.39 to 0.33)	
Self-concept	109	57		52			
Raw score			224.4 (218.94 to 229.86)		221.92 (216.14 to 227.69)	2.48 (-5.48 to 10.44)	0.53
Z-Score			-0.07 (-0.26 to 0.12)		-0.16 (-0.36 to 0.04)	0.09 (-0.19 to 0.36)	
Self-esteem	109	57		52			
Raw score			35.14 (34.03 to 36.25)		34.15 (32.98 to 35.32)	0.99 (-0.63 to 2.61)	0.22

© 2023 Migueles JH et al. *JAMA Network Open*.

Z-Score			0.46 (0.22 to 0.69)		0.25 (-0.002 to 0.50)	0.21 (-0.13 to 0.55)	
Composite score	109	57	0.04 (-0.19 to 0.26)	52	-0.04 (-0.27 to 0.2)	0.07 (-0.25 to 0.4)	0.65
Total mental health							
Composite score	109	57	0.02 (-0.19 to 0.23)	52	-0.02 (-0.24 to 0.2)	0.04 (-0.27 to 0.34)	0.81

Data analyses were conducted under the intention-to-treat principle, i.e., including all participants and imputing the missing data using predictive mean matching multiple imputations. Z-score values indicate how many standard deviations have the follow-up values changed with respect to the baseline mean and standard deviation. E.g., a 0.50 Z-score means that the mean value at follow-up is 0.50 standard deviations higher than the mean value at baseline indicating a positive change, with negative values indicating the opposite.

*Adjusted for baseline values.

†Higher values indicate lower mental health.

© 2023 Migueles JH et al. *JAMA Network Open*.

eFigure 1. Individual Change Distribution in the Cardiometabolic Risk Scores, Visceral Adipose Tissue, and Body Mass Index Affected by the Exercise Program

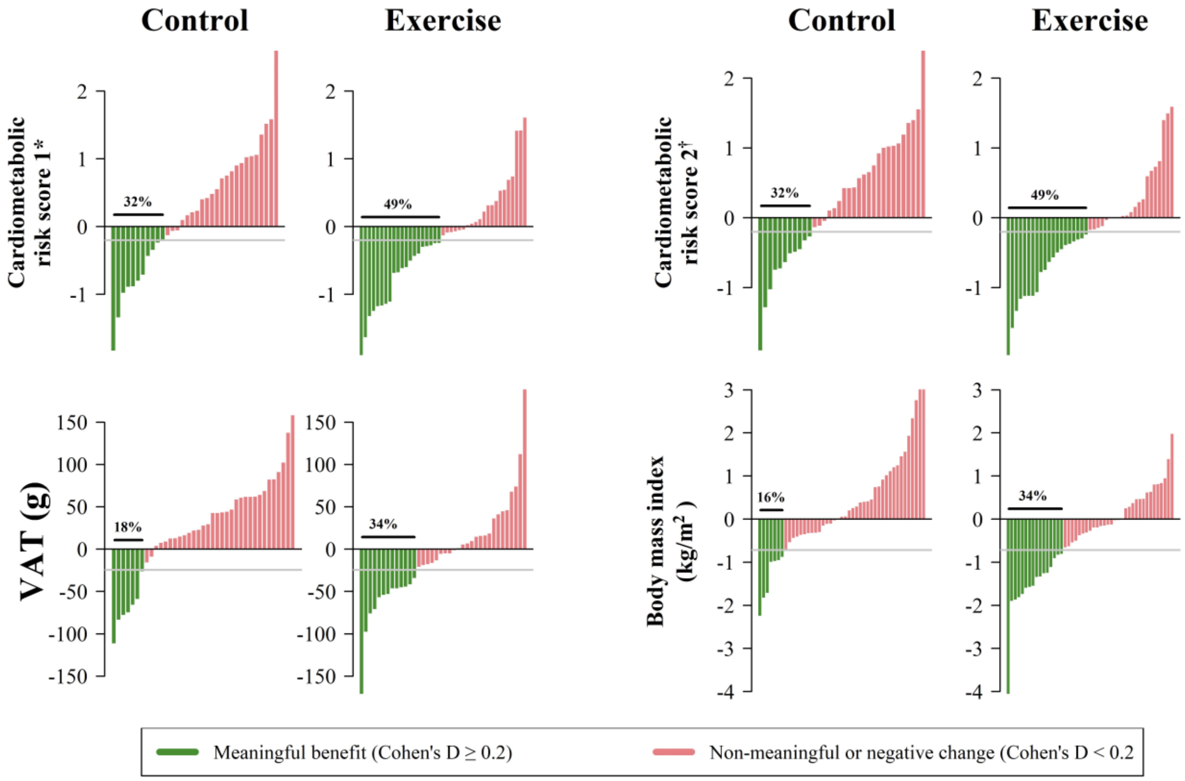

Data analyses were primarily conducted under the per-protocol principle, i.e., attending ≥ 70% of the sessions.
*Cardiometabolic risk score 1 was calculated as the age- and sex-normalized scores for HDL cholesterol, waist circumference, triglycerides, glucose, and the average of systolic and diastolic blood pressure based on European population reference values.[39]
†Cardiometabolic risk score 2 additionally included the CRF.
VAT: visceral adipose tissue.

© 2023 Migueles JH et al. *JAMA Network Open*.

eFigure 2. Change in Daily Activity Composition Induced by the Exercise Program as Measured With Hip- and Wrist-Worn Accelerometers

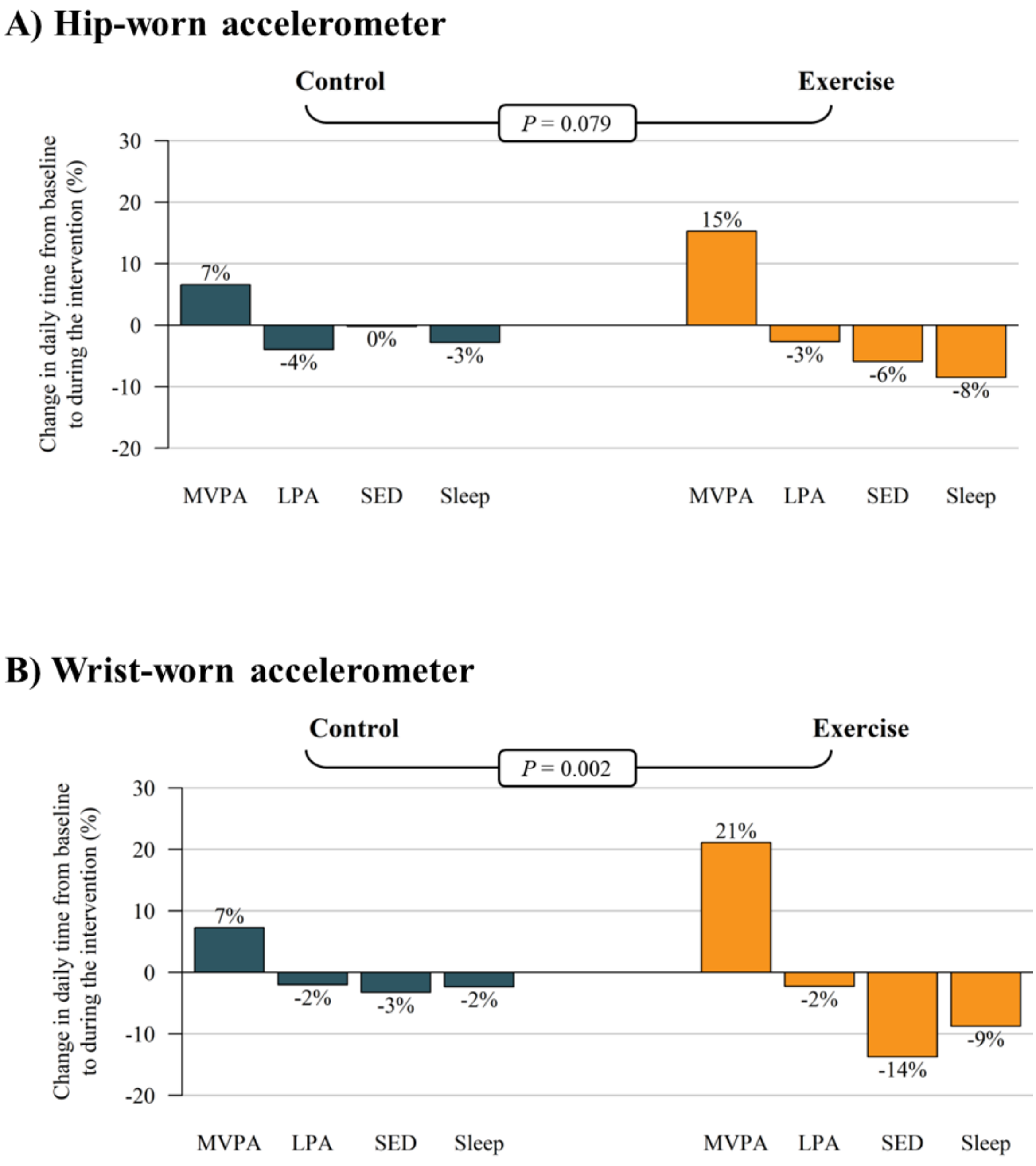

Data analyses were primarily conducted under the per-protocol principle, i.e., attending 70% of the sessions. Isometric log-ratios between each group's compositional mean and the overall compositional mean after centering the data at baseline and during exercise were calculated. *P* value from Hotelling's T-squared test for pair-wise comparison of multivariate means. MVPA: moderate-to-vigorous physical activity; LPA: light physical activity; SED: sedentary time.

© 2023 Migueles JH et al. *JAMA Network Open*.
