# Supplementary material for: Effects of an Exercise Program on Cardiometabolic and Mental Health in Children With Overweight or Obesity: A Secondary Analysis of a Randomized Clinical Trial
Source: JAMA Netw Open. 2023 Jul 27;6(7):e2324839. doi: 10.1001/jamanetworkopen.2023.24839 (PMC10375312; doi:10.1001/jamanetworkopen.2023.24839)
Supplement: Supplement 3. — Data Sharing Statement [file jamanetwopen-e2324839-s003.pdf]

Data Sharing Statement

Migueles. Effects of an Exercise Program on Cardiometabolic and Mental Health in Children With Overweight or Obesity. *JAMA Netw Open*. Published July 27, 2023. doi:10.1001/jamanetworkopen.2023.24839

Data
Data available: No
